# Orchestrating machine learning models in a swarm architecture for IoT inline malware detection

**DOI:** 10.1038/s41598-025-28859-w

**Published:** 2025-12-20

**Authors:** Muhammad Hanif, Ehsan Ullah Munir, Muhammad Maaz Rehan, Saima Gulzar Ahmad, Kashif Ayyub, Naeem Ramzan

**Affiliations:** 1 COMSATS University Islamabad, G.T Road, Wah Cantt, Islamabad, Pakistan; 2 School of Computing, Engineering and Physical Sciences, University of South of West Scotland, Paisley, Scotland, UK; 3 Department of Computer Science, University of Central Lancashire, PR1 2HE Preston, UK

**Keywords:** Internet of things, Machine learning, Active learning, Cyber security, FOG computing, ML model-based swarm, Intrusion detection system, Engineering, Mathematics and computing

## Abstract

The Internet of Things (IoT) represents a vast network of interconnected devices engaged in continuous data exchange, real-time information processing, and autonomous decision-making through the Internet. The pervasive presence of sensitive data on IoT devices highlights their indispensable role in our daily lives. The rapid evolution of Information and Communications Technology (ICT) has ushered in a new era of interconnected devices, reshaping the computing landscape. With the expanding IoT ecosystem, cyberspace has become increasingly susceptible to frequent cyber threats. While IoT devices have greatly simplified and automated daily tasks, these devices have simultaneously introduced significant security vulnerabilities. The existing inadequacies in safeguarding these smart devices have rendered IoT the most vulnerable entry point for potential breaches, posing a tempting target for malicious actors. In response to these critical challenges, our study introduces an innovative solution known as Swarm-based Inline Machine Learning (SIML). This approach leverages the coordinated data processing capabilities of a swarm to effectively address and counter emerging malware threats. SIML represents a divergence from conventional standalone threat detection systems, offering a promise of more robust, distributed, and end-to-end security solutions for IoT environments. This approach significantly reduces the risk of malicious exploitation of IoT devices for launching cyber-attacks. The effectiveness of our proposed method was validated through rigorous testing using the UNSW-NB15 dataset. The results are compelling, boasting an impressive accuracy rate of 93.7% and a precision rate of 95%, achieved through the application of the Gradient-Boosting Tree algorithm under the proposed framework. Our comparative analysis reveals that the Gradient Boosting algorithm outperforms traditional methods without compromising efficiency when deployed in an inline setting. Furthermore, the proposed method has been benchmarked against the BoT-Iot and Edge-IIoTset datasets, and outperformance is noted with a minor degradation at higher throughput. This innovative approach not only enhances security in IoT but also paves the way for a safer and more resilient digital future.

## Introduction and background

### Introduction

The Internet of Things (IoT) represents a significant evolution, signifying a remarkable stride in the realm of technology. It seamlessly amalgamates traditional computer science facets, including networking, mobile computing, and software engineering, with the realm of electronics, encompassing actuators, communication protocols, sensors, and embedded technology. This fusion of scientific and technological domains opens up a plethora of possibilities to enhance human well-being. As technology advances, it ushers in novel approaches that outshine conventional methods, and IoT emerges as a comprehensive and inventive solution to the connectivity challenge^1^. IoT heralds an era where electronic devices transform into intelligent, interconnected entities. These connected devices offer consumers a lifestyle that is not only more convenient but also highly efficient. For instance, one can now effortlessly order meals from the comfort of their bed and employ virtual assistants to streamline daily tasks. Nevertheless, the ubiquity of these technological marvels has also exposed individuals to the inherent risks of the internet environment^2^.

IoT, being a revolutionary 21st-century technology, remains in a perpetual state of research and development, finding widespread applications across diverse domains. However, this pervasive integration gives rise to significant apprehensions, primarily in the domain of security, concerning both organizations and researchers. IoT devices transcend the boundaries of the conventional internet, now extending connectivity to virtually every conceivable object. This remarkable expansion empowers individuals to connect and remotely manage an astonishing array of devices^1^. Nevertheless, this extensive adoption of IoT devices over the internet, combined with their constrained processing capabilities, renders these devices susceptible to cyber attacks. Another pivotal vulnerability factor lies in the design of communication protocols, which frequently overlook security considerations and cybersecurity prerequisites, resulting in a disconcerting prevalence of network breaches and privacy infringements.

The Internet of Things (IoT) has emerged as a paradigm-shifting technology, weaving a network of billions of interconnected devices into the fabric of our daily lives and critical infrastructures. While this hyper-connectivity drives unprecedented innovation and efficiency, it also introduces a vastly expanded attack surface. Unlike traditional computing systems, IoT devices are often resource-constrained, deployed in physically insecure locations, and designed with a focus on functionality over security. This combination of factors makes the IoT ecosystem a prime target for a wide range of cyber-attacks, from large-scale botnets to stealthy data exfiltration^3^.

Conventional security measures, such as signature-based intrusion detection systems (IDS) and centralized, cloud-based analytics, are ill-suited for the unique challenges of the IoT. Signature-based methods are ineffective against novel and zero-day attacks^4^, while centralized models introduce significant latency, making real-time threat mitigation nearly impossible. Furthermore, the sheer volume of data generated by IoT devices can overwhelm centralized systems, creating a bottleneck for analysis. There is a critical need for a new security paradigm that is decentralized, adaptive, and capable of operating in real-time at the network edge.

To address these challenges, this paper introduces a Swarm-based Inline Machine Learning (SIML) framework for IoT malware detection. SIML leverages the principles of swarm intelligence to create a collaborative, decentralized network of lightweight machine learning models that operate directly within the network traffic flow. This inline approach enables the real-time detection and mitigation of threats before they can cause harm. By distributing the detection capabilities across a swarm of agents, SIML eliminates single points of failure, enhances resilience, and provides a scalable solution for securing large and dynamic IoT networks. This study demonstrates that the proposed SIML framework, particularly when implemented with a Gradient Boosting Tree algorithm, achieves high accuracy and precision in detecting a wide range of attacks, offering a significant advancement over traditional methods.

### Background

Malware detection has been a critical topic since the advent of the Internet. Over time, as technology has evolved, so have the methods for detecting malware. However, malware development and deployment techniques have also become increasingly sophisticated. This ongoing evolution necessitates dedicated efforts to safeguard assets from cyberattacks.

A disconcerting revelation from a Palo Alto security survey report underscores a critical issue: a staggering $98\%$ of the internet traffic generated by IoT devices remains unencrypted^5^. This alarming statistic reveals that attackers who successfully breach the initial line of defense, often through phishing attacks, can intercept unencrypted network traffic. The attacker proceeds to gather personal or sensitive data, which is then manipulated and exploited on the dark web for their illicit gains. In response to this escalating threat, security service providers resort to sandboxing and signature-based threat detection solutions to scrutinize malware, to mitigate evasive attacks^6^. Unfortunately, these conventional methods take a toll on user usability and disrupt workflows by retaining research files, scanning or modifying content, and processing an extensive number of files. Moreover, these strategies grapple with a critical limitation: conventional methods can only defend against new attacks after the attacks have been detected or have already caused damage to an organization, a scenario commonly referred to as a zero-day attack.

In the domain of cyber defense, Machine Learning (ML) has assumed an increasingly pivotal role. Research efforts have been dedicated to leveraging ML for a diverse range of applications, spanning from antivirus systems to the identification of malicious scripts. It’s essential to recognize that even the slightest variations in our data can have far-reaching implications for model accuracy and downstream processes that rely on these model predictions. ML modeling is deliberately crafted to unearth non-linear dependencies within input data^7^. However, within the realm of cyber defense modeling, this deliberate quest for non-linearity introduces an inherent dilemma: (1) the imperative need to adapt models to the evolving landscape of threats over time, and (2) the realization that adjustments to models may yield unforeseen consequences that must be diligently addressed.

The progression of IoT technology has ushered in a transformative era, with IoT applications now constituting a significant $30\%$ of devices within corporate networks^8^. This proliferation of IoT devices offers valuable insights derived from the vast pool of data these devices collect, enabling real-time decision-making and accurate predictions. IoT plays a pivotal role in automating processes, enhancing supply chain management, and ensuring compliance with regulatory standards. It achieves this by facilitating the seamless integration of Information Technology (IT) and Operational Technology (OT) systems, resulting in substantial reductions in both capital and operational costs. Furthermore, IoT serves as a central enabler of digital innovation within organizations, harboring the potential to elevate employee productivity, enhance corporate performance, and strengthen profitability^9^.

**Fig. 1:**
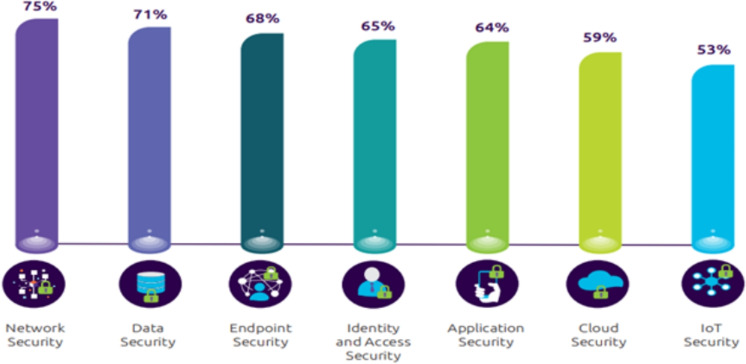
An overview of security aspects and contribution of AI in cybersecurity^10^.

The depiction of security aspects and their contribution to the broader cybersecurity landscape is revealed through the research conducted by the Capgemini Research Institute^10^. With the exponential surge in network traffic, the task of identifying anomalies in behavioral patterns poses an escalating challenge for cybersecurity analysts. The study conducted by the Capgemini Research Institute has arrived at a significant finding: a substantial $53\%$ of security threats can be attributed to vulnerabilities in IoT devices. This research also highlights the alarming and continuous increase in cyber-attacks attributable to the vulnerability of IoT devices, as indicated in Fig. 1 .

This innovative approach to cyber-attack detection sets itself apart from traditional methods by integrating intelligence and intelligent solutions. It leverages lightweight inline firewall-level models to proactively thwart a wide range of impending attacks, complemented by a Fog-based ML solution that rapidly provides signatures to ML-powered firewalls^11^. It places special emphasis on deploying in-line supervised ML techniques to enhance both host-based and network-based security solutions within the IoT environment. The system operates through three key components: defining IoT device behavior to profile connected devices, detecting malicious network packets during potential cyber attacks, and classifying the specific type of cyber-attack in progress. This comprehensive approach offers robust defense against a multitude of threats in the IoT landscape.

### Problem statement

IoT devices play a crucial role in daily life, and the recent evolution of smart IoT functionalities has heightened the need for hyper-connectedness^3^. Unfortunately, these hyper-connected devices are vulnerable to malicious agents^12^, with the IoT device itself being the most exploited vulnerability^13^. Zero-day attacks^14^, DDoS^15^, Botnets^16^, and physical security threats^17^ are prevalent challenges faced during the security of IoT networks. An ML-based solution is designed to train on static data and requires updates^18^, which itself poses a threat when not timely updated. These research gaps require a proactive solution. In order to address these challenges of new cyber-attacks detection^19^, adaptive knowledge update^20^ supported and a continuously available system, a Swarm-based inline ML (SIML) mechanism is proposed.

A high-performance ML-based system is inspected based on the detection rate, the size of data being processed, and the detection rate. To achieve higher performance, the framework should be optimally constructed. By definition, the proposed framework can be defined as: *D* is the Malware Detection Rate, *R* is the Signature Generation Rate, *T* is the Malware Traffic, *P* is the Policy Enforcement Efficiency, and *M* represents the Malware Generation Rate. Then the mathematical equation can be formulated for the malware detection rate.

1 $$\begin{aligned} D = \frac{R \cdot \int _{0}^{T} \sigma (t) \, dt \cdot \gamma (P)}{M} \end{aligned}$$

2 $$\begin{aligned} D = f\left( \frac{R \cdot T \cdot P}{M}\right) \end{aligned}$$

Where, D: Malware Detection Rate, R: Signature Generation Rate, T: Malware Traffic over time, represented by, $\sigma (t)$, the traffic function over time t, P: Policy Enforcement Efficiency, modeled as a function, $\gamma (P)$ that reflects its impact, and M: Malware Generation Rate.

3 $$\begin{aligned} \frac{dx_i(t)}{dt} = -\alpha x_i(t) + \beta \sum _{j \in \mathcal {N}_i} (x_j(t) - x_i(t)) + \gamma u_i(t) \end{aligned}$$

Where:

$x_i(t)$: State of the $i$-th node (load, suspicion level) at time $t$

$-\alpha x_i(t)$: Self-stabilizing term driving toward equilibrium

$\beta \sum _{j \in \mathcal {N}_i} (x_j(t) - x_i(t))$: Consensus dynamics with neighbors $\mathcal {N}_i$

$\gamma u_i(t)$: External input from traffic and controller directives

The Eq. (1) incorporates the integration of malware traffic over time and the impact of policy enforcement, highlighting the dependencies and relationships among these variables in optimizing detection. This framework emphasizes the balance between detection efficiency and the evolving nature of threats. Whereas, Eq. (2) dictates the effectiveness of the proposed method, where the rate of malware detection should remain higher than the rate of malware generation. The Eq. (3) provides a mathematical representation for analyzing the swarm’s stability and convergence properties under dynamic conditions, addressing the reviewer’s concern about the need for formal analysis beyond your initial high-level equations.

Traditional security measures are ill-suited for IoT networks due to the unique constraints of limited device resources, the critical need for real-time threat detection, a dynamically evolving attack surface, and immense scalability requirements. Current Machine Learning (ML) solutions exacerbate these issues by often operating offline or as vulnerable, monolithic centralized systems. This creates a significant research gap, underscoring the critical need for a security framework that is simultaneously lightweight, inline, adaptive, and resilient to effectively protect IoT ecosystems.

### Research Contributions

Swarm-based Machine Learning (SML) deploys multiple ML models in a distributed environment, enabling improved system availability, fault tolerance, and enhanced performance through collaborative prediction and aggregation. In-line ML integration involves deploying ML models directly within the network traffic flow, typically at the firewall level^21^. This allows for real-time analysis of network traffic, enabling the detection and blocking of malicious activity before it can impact the network. SML techniques can be leveraged to enhance the performance and robustness of In-line ML systems, providing a more resilient and effective defense against sophisticated cyber threats.

This research study explores the integration of swarm-based in-line ML for enhanced malware detection in IoT networks. By leveraging advanced machine learning techniques, we aim to improve the ability of firewalls to identify and mitigate emerging threats in real-time, ensuring the security and resilience of the evolving IoT ecosystem. The contributions of this research study are as follows:

- To design a robust and enhanced framework for detecting cyberattacks in IoT-based networks using an ML model.
- To establish a swarm-based inline ML mechanism for strengthening cybersecurity at the firewall level within IoT networks to effectively counter unknown threats.
- To demonstrate the utility, feasibility, and performance assessment of ML model-based swarm approach using supervised ML classifiers.

The research study is structured as follows: section “Related research work” offers an in-depth exploration of the existing literature. Section “Proposed framework and methodology” delves into the proposed research methodology, elucidating the experimental setup and the tools and technologies harnessed for the study. In section “Result and discussion”, a comprehensive examination of the results takes place, encompassing a thorough discussion of the core attributes, processing methodologies, and a meticulous analysis of the outcomes. Finally, section “Conclusion and future work” serves as the study’s conclusion, summarizing the entirety of the research and shedding light on potential avenues for future research extensions.

## Related research work

In recent years, the utilization of IoT devices has witnessed a substantial surge. Their pervasive connectivity facilitates seamless collaboration, knowledge exchange, and intelligent decision-making in conjunction with other technologies^22^. The Internet of Things (IoT) encompasses the interconnection of physical objects, vehicles, and various structures equipped with hardware, software, sensors, actuators, and network interfaces. This inter-connectivity empowers these entities to collect and disseminate data^23^. In essence, IoT endows physical entities with the intelligence to sense and transmit information from their surroundings. Its applications span across various domains, simplifying life, enhancing competitiveness, and reducing costs for businesses. The scope of IoT remains extensive, with its transformative influence permeating every sector of society and industry, ushering in significant disruptions^24^.

A layered model is introduced by^25^ for detecting anomalies in networked IoT devices. The proposed model, known as Profile and Hierarchical Incrementally-Based Anomaly Detection (PHICAD), employs a lightweight ML algorithm. The research involves an evaluation of the PHICAD algorithm using two distinct datasets, ISCX-IDS-2012 and CIC-IDS-2017. The results indicate a commendable performance, with an F1 score of $81\%$ for the former dataset and an even more impressive F1 score of $91\%$ for the latter, showcasing the efficacy of the PHICAD algorithm in anomaly detection for IoT networks.

Bi-LSTM neural network-based trust evaluation method is proposed^26^ as a mechanism for trust evaluation of IoT devices by learning network behavior patterns and time-dependent relations. This approach calculates the trust value using the Bi-LSTM model, achieving an impressive accuracy of $93.8\%$ and an R-square value of $88\%$. On the other hand, research study^27^ employed feature reduction techniques, specifically Principal Component Analysis (PCA) and Linear Discriminant Analysis (LDA), along with a two-tier classification system to detect various types of attacks, including Denial of Service (DoS), User to Root (U2R), Remote to Local (R2L), and others. The research study utilized supervised ML algorithms such as Naive Bayes (NB), Support Vector Machine (SVM), Multilayered Perceptrons (MLP), Decision Tree J48 (J48), and Zero Rule (ZeroR). The research work reduced the feature count from 35 to 2 features in the NSL-KDD dataset. The system demonstrated a detection accuracy of $84.82\%$ as measured by the F-measure, effectively identifying anomalous traffic on the network.

In a research study^28^ author explored the application of various ML algorithms, including K-Nearest Neighbor (KNN), Artificial Neural Network (ANN), Decision Tree (DT), Naive Bayes (NB), Support Vector Machine (SVM), Random Forest (RF), and Linear Regression (LR), using the Bot-IoT dataset. The research assessed these algorithms based on performance metrics such as accuracy, F1 score,log-loss, and recall. Notably, the study found that RF outperformed other algorithms in the detection of Distributed Denial of Service (DDoS) attacks, achieving an impressive accuracy rate of $99\%$. RF also excelled in binary classification problems, particularly in the case of HTTP traffic. For multi-class classification settings, KNN demonstrated superior performance, surpassing the $99\%$ accuracy mark, while RF performed slightly lower, with a $4\%$ lower accuracy rate than KNN. This suggests that the choice of algorithm can significantly impact the accuracy of intrusion detection in IoT networks.

Three-layered lightweight standalone Intrusion Detection Systems (IDS) are introduced in^29^ for IoT device-based networks. The research study utilized supervised ML techniques and was evaluated on a custom-built dataset. The authors employed various ML algorithms, including Naïve Bayes, J48, Simple Logistics, SVM, MLP, and Random Forest, to classify IoT network traffic. The study aimed to classify IoT device behavior and profiles for both normal and abnormal traffic generators. To assess its effectiveness, the researchers utilized a smart home test bed consisting of eight commonly used IoT devices available on the market. The proposed system demonstrated remarkable performance in detecting various threats, including DoS attacks, man-in-the-middle attacks, spoofing, recognition, and replay attacks within the IoT network. The results were exceptional, with the system achieving an F-measure score of $98\%$. Additionally, the study made significant strides in the automated recognition of IoT devices from network activities. It effectively categorized the activities as benign or malicious based on network traffic features, further enhancing security within the IoT environment.

AI-based monitoring agent is proposed by^30^, with capabilities of network traffic pattern recognition using supervised ML. The research is conducted on comprehensive research on IoT devices, focusing on system security mechanisms. The researchers utilized the NSL dataset for validation of the proposed method and employed data mining methodologies to filter and process the data. The primary objective of the study was anomaly-based Intrusion Detection for IoT. Binary classification was carried out using the Support Vector Machine (SVM) method. The results of the research were highly promising, with a detection accuracy of 99.71 percent. The system demonstrated the capability to effectively detect a range of attacks, including DDoS, U2L, L2R, and Probe attacks. This study contributes significantly to enhancing the security of IoT systems. The proposed method fails to exhibit the ML maintaining strategy for long-standing against the new attacks. The proposed method did not cover the ML model sustainability in a real environment.

An ML algorithm-based model was developed by^1^ to identify and counteract botnet-based attacks on IoT networks using the BoT-IoT dataset. The study employed linear regression, logistic regression, KNN, and SVM models, achieving F-measures of 98.0%, 99.0%, and 99.0%. Results show that the latency of the ML model is not considered, and the real-time processing capability of the model was also not tested. In the research conducted by^31^, the objective was to create a trustworthy, optimization-based environment to reduce security risks in IoT networks. The study utilized a custom dataset and employed the Adaptive Tunicate Swarm Algorithm (ATSA). While the establishment of a trustworthy environment was verified, specific results for further analysis were not provided.

The study conducted by^11^ aimed to detect cyber attacks within IoT networks through an anomaly-based approach. To facilitate their research, the authors utilized the BoT-IoT dataset^32^ and applied a PCA-based feature reduction technique to streamline the dataset. This reduction process resulted in a final model with only seven lightweight features. The proposed approach proved to be highly effective in detecting various cyber attacks, including DDoS, DoS, Reconnaissance, and information theft assaults. To make comprehensive comparisons, the researchers evaluated various ML algorithms, including KNN, LR, SVM, MLP, DT, and RF. Notably, the study revealed that the Random Forest (RF) algorithm performed exceptionally well, achieving a remarkable accuracy of 99.9%. Furthermore, RF exhibited a shorter test training time compared to the other algorithms. This research study provides valuable insights into the efficient detection of cyber attacks in IoT networks.

Cyber-attack detection in Cyber Physical System (CPS) is explored in^33^, intending to enhance attack detection in an IoT-based CPS environment using a swarm-based feature selection algorithm with the NSL-KDD dataset. The researchers utilized the Enhanced Chicken swarm optimization (ECSO) combined with Recurrent Neural Network (RNN), achieving an accuracy of 99.2% and an ROC score of 92.0%. However, the real-time detection of attacks as well strategy for keeping the ML model up to date is not considered in the research study.

The study conducted by^34^ aimed to enhance IoT security by employing network profiling and ML. The Cyber-Trust test-bed, utilizing normal and malicious network traffic from a smart home environment, was the chosen dataset. A MobileNet CNN (MobileNetV3) was employed, achieving an accuracy of 98.35%, with a low false-positive rate of 0.98%. However, it’s important to note that the trust profiling mechanism was not tested, leaving a potential gap in understanding the system’s overall effectiveness.

In the research study^35^, conducted with the objective of detecting fraudulent traffic and enhancing IoT ecosystem stability. The study utilized the IoT-23 dataset and applied a combination of Long Short-Term Memory (LSTM) and Generative Adversarial Network (GAN)-based methods. The achieved accuracy was 97%, showcasing the effectiveness of the approach. Nevertheless, it’s worth noting that the method was tested on a limited dataset, indicating the need for further validation on diverse datasets to ensure broader applicability and robustness. In a study^36^ author proposes machine-learning-based systems for detecting malicious traffic in IoT networks by focusing on Darknet traffic, achieving an accuracy of 99.5% with a bagging decision tree ensemble. While^37^ noted that a hybrid deep learning model, including a CNN and a long-term short memory neural network, performs well for detecting DDoS attacks in a real environment.

Swarm-based inline ML techniques have been proposed for cyber-attack detection in IoT devices. These techniques utilize swarm intelligence algorithms, such as Grey Wolf Optimization (GWO), to optimize the hyperparameters of ML models and find the most relevant features for detecting IoT botnet attacks^38^. One Class Support Vector Machine (OCSVM) is a powerful algorithm used for anomaly detection in IoT botnet attacks^39^. Another approach involves using a hybrid feature reduction technique that combines different feature ranking methods and ML algorithms, such as RF, KNN, and XGBoost, to detect cyber-attacks in IoT networks^40^. Bayesian optimization Gaussian Process (BO-GP) algorithm and decision tree (DT) classification models have also been proposed for effective and efficient attack detection in IoT devices^41^. ML algorithms, such as RF and KNN, have shown promise in detecting malicious and anomalous data in IoT systems^16^.

The preceding research has extensively explored the potential of modern ML and deep learning techniques in bolstering IoT security. A crucial aspect of this research involves utilizing ML classifiers, which require a suitable dataset fine-tuned to the specific conditions for which the classifier is being trained. Fine-tuning a classifier is a critical task that directly impacts the performance of the ML model. Ongoing research trends focus on enhancing performance through fine-tuning, feature reduction, and engineering methods. However, the situation becomes more challenging in compute-constrained networks, where research on lightweight methods with improved performance is essential. Researchers often attempt to replicate IoT scenarios to create an IoT-specialized IDS due to the unavailability of datasets with IoT scenarios. Understanding feature relevance for classification is crucial before implementing ML classifiers, especially in the absence of IoT-specific datasets. The literature review highlights that ML and deep learning algorithms with advanced feature engineering and reduction strategies outperform traditional intrusion detection and network traffic analysis methods. Nevertheless, for resource-constrained environments like IoT, lightweight solutions based on conventional ML algorithms are more suitable.

### Gaps in literature

While existing studies demonstrate high accuracy, they often lack real-time adaptability and swarm-based collaboration. For instance,^26^ achieves 93.8% accuracy but does not address synchronization overhead. Our work fills this gap by introducing swarm intelligence for fault tolerance.

Recent advancements in deep learning, such as^42^, show promise in industrial IoT but suffer from high latency. In comparison, SIML’s hybrid approach outperforms in resource-constrained environments.

While existing research has explored the use of machine learning for IoT security, several critical gaps remain. Many proposed solutions are designed for offline analysis and are not suitable for real-time, inline deployment. Furthermore, the resilience of these models to adversarial attacks and their performance in resource-constrained environments are often not evaluated. Finally, most studies focus on centralized models, which represent a single point of failure and do not offer the high availability required for critical IoT applications. Our work directly addresses these gaps by proposing a swarm-based, inline framework that is designed for resilience and efficiency.

Despite the extensive research in ML-based IoT security, several critical gaps persist in the literature. A significant body of work focuses on developing highly accurate detection models, but often overlooks the practical constraints of real-world IoT deployments. Our review of the literature reveals the following key limitations:

- **Lack of Inline and Real-Time Solutions:** Many proposed systems are designed for offline analysis of captured network traffic^43^. While useful for forensic purposes, these approaches cannot prevent attacks in real-time. There is a scarcity of research on lightweight, inline models that can be deployed directly on network gateways or firewalls.
- **Centralized Architectures:** The majority of existing solutions rely on a centralized architecture, where data is sent to a powerful server or cloud for processing. This creates a performance bottleneck, introduces latency, and represents a single point of failure. Decentralized and distributed approaches, while acknowledged as a promising direction, are not yet widely explored.
- **Limited Evaluation of Scalability and Resilience:** While many studies report high detection accuracy, few provide a rigorous evaluation of their system’s scalability to large networks or its resilience to node failures and adversarial attacks. The performance of a model in a controlled lab environment may not translate to a dynamic and hostile real-world setting.
- **Neglect of Resource Constraints:** The computational cost of the proposed models is often not adequately considered. Many deep learning models, while powerful, are too resource-intensive for deployment on edge devices^44^.
- **Assumption of Labeled Data Availability:** Most supervised learning models require large amounts of labeled data for training. However, obtaining labeled data for new and emerging IoT threats is a significant challenge. Techniques that can learn with limited labeled data, such as semi-supervised or federated learning, are underexplored^45^.

The proposed SIML framework is designed to directly address these gaps. By adopting a swarm-based, inline architecture, SIML provides a solution that is decentralized, scalable, resilient, and optimized for real-time performance in resource-constrained environments.

In conclusion, the increasing prevalence of IoT devices has revolutionized connectivity and decision-making across various domains. The detailed literature overview presented here underscores the evolving landscape of IoT security. Several studies have proposed innovative approaches, such as anomaly detection models and trust evaluation methods, to fortify IoT network security. However, challenges persist, including high processing times, model complexity, and the need for real-time attack detection. The significance of choosing appropriate ML algorithms is evident, with varying performance observed across different models. While many proposed solutions showcase high accuracy rates, considerations like model sustainability and adaptability to new attacks warrant further exploration in future research efforts. Overall, the dynamic nature of IoT security demands continual innovation and comprehensive solutions that address emerging challenges.

The cybersecurity landscape has evolved from an offensive (security-first) to a defensive (resilience-first) approach, leveraging advanced technologies with effective predictive analysis capabilities. Ongoing research aims to augment ML model performance using novel approaches, primarily focusing on optimizing the pre-process profile of the ML model to enhance performance. This research study addresses this gap by introducing a novel approach: Swarm-based configuration of ML models. This approach aims to achieve resistance against deception attacks and reduce system downtime due to adaptive training and deployment strategies for ML models. The subsequent sections provide a detailed discussion on the proposed research methodology, framework, and system architecture.

## Proposed framework and methodology

An ML-based solution is being used by the industry in an out-of-band-detection configuration, which increases the overhead and expands the response time. An in-line ML-based intrusion detection system is a critical task; to achieve that conventional IoT network architecture needs to be redesigned. IoT architecture has three main levels: sensor, network, and layer of application^30^. The perception layer is everything from the sensors to the collection of information. Misleading physical attack on sensor equipment, illegal access to equipment, etc. The network layer connects sensors and actuators via Wireless (WiFi, LAN, 3G, 4G)^46^ devices and gateways. Therefore, the most common attacks on this layer include DoS and DDoS, information theft, data collection, gateway assaults, attacks on routers, etc. An anomalous traffic detection or prevention system from the network is essential to overcome these attacks. The research study^17^ concludes that Static Analysis (SA) of IoT systems is a popular method that has repeatedly been proven to be effective. ML-based intelligent frameworks are the need of the day, which contain a whole set of elements for the construction of methods of nonlinear variations of SA for complex IoTs, while being amenable to complete automation.

### What makes an IoT device vulnerable to cyber attacks?

The rise of smart devices in the digital revolution poses security challenges in IoT networks. Challenges include the heterogeneity of devices, the vast volume of interconnected devices, dynamic network reconfiguration, vulnerability to various attacks, short-range ad hoc networks, low-latency and high-reliability requirements, cost and energy consumption considerations, and the imperative need for robust security and privacy protection, especially in sensitive domains like healthcare^47^. Intelligent, real-time decision-making is essential for optimal functionality in diverse IoT applications.

The Intrusion Detection System (IDS) is to uncover malicious activity without a conventional firewall^33^. The IDS continuously monitors the network and seeks suspicious activities throughout the network^12^. By quickly scanning the network packet, IDS acts on the network layer of the protocol stack. IDS are classified as Anomaly-based Intrusion Detection Systems (AIDS) or Signatures-based Intrusion Detection Systems (SIDS)^28^. SIDS are a robust method to detect known attacks; however, it is useless to any unanticipated attack^25^. A dynamic network, such as IoT, cannot thus rely on the signature-based IDS. AIDS identifies the anomalies by use of ML and is considered efficient for detecting unknown attacks. Such systems improve performance with continuous learning from new knowledge gained over time. It is therefore ML-based systems supposed to be more productive for a diverse IoT ecosystem.

### Swarm intelligence vs ML model-based swarm

Swarm intelligence is a concept inspired by the natural behaviors of groups such as ants, bees, and birds, where members work collectively and respond dynamically to changes without centralized control. Extending this idea to machine learning, ML models based on swarm introduce a novel approach where ML models are organized as a swarm. This configuration enhances knowledge delivery by leveraging distributed, coordinated, and collective learning processes.

ML model-based Swarm offers significant advantages for cybersecurity:

- Adaptability: Swarm-based systems continuously learn and adapt to evolving threats, ensuring up-to-date defenses.
- Decentralized Response: Unlike traditional centralized defense systems, swarm intelligence distributes detection and response across nodes, reducing single points of failure and increasing robustness.
- Scalability: Swarm-based models effortlessly scale to accommodate large and complex networks, making them suitable for organizations of any size.

In a swarm-intelligent system, each device or network node functions as an autonomous agent, actively scanning for suspicious activities or potential threats. When a node identifies an anomaly, it communicates with others in the network. Together, these nodes collaborate to form a unified and comprehensive response, coordinating actions in real-time without the need for a central command structure. This decentralized and adaptive approach significantly enhances the resilience and efficacy of cybersecurity frameworks.

### Feature selection and reduction

We employed correlation-based feature selection specifically to balance model performance with the stringent computational constraints of inline IoT security. As our results demonstrate (e.g., 94.3% F1-Score on UNSW-NB15), this method proved highly effective and sufficient for achieving robust detection accuracy. While we acknowledge that advanced techniques like Particle Swarm Optimization (PSO) and Grey Wolf Optimization (GWO) can offer robust feature sets in general, their significant computational complexity, extended convergence time, and high-power consumption are prohibitive for our target environment. These factors directly contradict the core requirement of low-latency, high-efficiency processing essential for real-time malware detection in resource-limited IoT edge devices. Therefore, the choice of a simpler, more efficient feature selection method was a critical design decision to ensure the practical viability of our proposed swarm architecture.

### System architecture

**Fig. 2:**
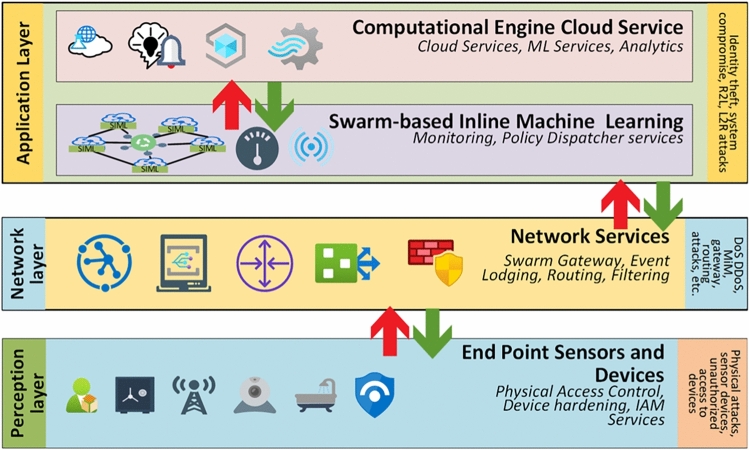
Communication layers design concept for integration of swarm-based inline ML for intrusion detection in IoT-based network, highlighting data flow, model coordination, and interaction between swarm agents and the firewall.

The proposed methodology utilizes an ML model-based Swarm to provide easy access to the nearest available traffic processing machine for malware detection. To achieve this capability Fog-based computing architecture is proposed. Fog computing architecture, being the nearest to the edge device, provides an efficient addition to the device’s ability to work properly. This approach is proposed to overcome the drawbacks of processing power, longer time delay, higher bandwidth, and higher communication cost to process the data at a central processing server. The system architecture is illustrated in Fig. 2. The application layer consists of data analytics to visualize the traffic on the network, a control service dedicated to dispatching and updating the blocking firewall policies to the Fog service providing endpoints. The final ML model was deployed in multiple containers in the initial stage and gradually updated the blocking policy with the aim of time as the requirement arose through the policy dispatcher service.

The network layer consists of the event processing and recording model as a swarm gateway and directly communicates with the application layer for any anomaly in the traffic. The network layer works as the input layer for the application layer, and the traffic is controlled at the same layer using a swarm gateway. The swarm gateway implements the policies generated by the system to control the traffic. The perception layer consists of the IoT devices working in the real-time environment and generating and utilizing the traffic coming and going to the network layer. Due to the lower processing capability of IoT devices, the decency from a privacy and security point of view cannot be relied upon. Therefore inline ML approach is proposed to ensure real-time traffic processing for edge devices.

The proposed framework’s effectiveness is modeled using Eqs. (1) and (2). Equation (1) is derived from the integration of malware traffic over time, where $\sigma (t)$ represents the traffic function, and $\gamma (P)$ models policy enforcement efficiency. The complexity analysis shows that the detection rate D scales linearly with R and P but inversely with M, indicating stability under dynamic attacks. For stability, we ensure $D > M$ through adaptive thresholds. The mathematical formulation presented in Eqs. (1), (2), and (3) provides a high-level conceptual model for the SIML framework. It is intended to illustrate the key factors influencing the malware detection rate, rather than serving as a precise predictive model. In this conceptualization, the detection rate (*D*) is a function of the signature generation rate (*R*), the volume of malicious traffic (*T*), the efficiency of policy enforcement (*P*), and the rate of new malware generation (*M*). The model highlights the dynamic nature of the problem, where the effectiveness of the defense system depends on its ability to generate and enforce policies at a rate that outpaces the emergence of new threats. A more rigorous analysis of the system’s performance, including latency and overhead, is presented in the experimental sections of this paper.

### Machine learning model-based swarm strategy

**Fig. 3:**
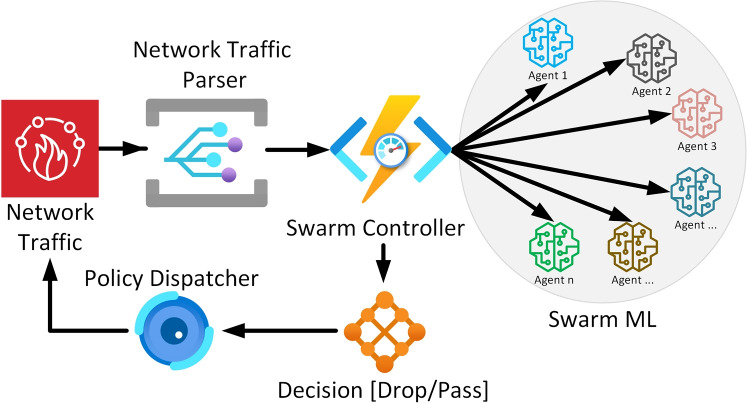
Swarm-based deployment of Machine Learning models for collaborative working and knowledge sharing.

Machine learning algorithms are increasingly employed in various configurations to achieve optimal outcomes. However, the training of ML models necessitates data and computational resources, and maintaining and updating these models in a production environment is considered a critical task^48^. The emergence of zero-day attacks presents a contemporary challenge to both research and industry, leading to severe data breaches and financial losses. The proposed swarm intelligence framework employs a distributed ensemble of machine learning models coordinated through a centralized controller that facilitates collaborative decision-making while ensuring operational security. ML model-based swarm configuration is a novel technique that involves connecting ML models in a swarm fashion, enabling them to process data collaboratively. The monitoring system assesses the swarm’s performance, allowing for the real-time updating of any underperforming models. This strategic approach provides the flexibility to update ML models without disrupting the efficiency of the production system. Figure 3 shows the ML model-based Swarm model configuration in an SDN-based IoT network. As the SDN-based network provides better control over the network traffic, therefore, deploying the ML model in an inline configuration would augment the security control of the network.

The theoretical foundation of our approach builds upon ensemble consistency and Byzantine fault tolerance principles.

4 $$\begin{aligned} \hat{y} = \Phi \left( \sum _{i=1}^{N} w_i \cdot f_i(x) \right) \end{aligned}$$

Where $f_i(x)$ represents the prediction of the $i$-th model in the swarm, $w_i$ denotes the dynamically adjusted weight based on model confidence and historical performance, and $\Phi$ is the aggregation function that ensures consensus. The convergence properties of this system can be analyzed through the lens of distributed consensus algorithms, where the controller acts as the consensus coordinator.

The consistency across heterogeneous model versions is formally guaranteed through the following mechanism:

5 $$\begin{aligned} \lim _{t \rightarrow \infty } \mathbb {P}\left[ \left| f_i^{(t)}(x) - f_j^{(t)}(x)\right| > \epsilon \right] \le \delta \end{aligned}$$

where $f_i^{(t)}$ and $f_j^{(t)}$ represent different model versions at time $t$, $\epsilon$ defines the acceptable prediction variance, and $\delta$ represents the maximum probability of divergence. This bounded divergence ensures that while models maintain diverse knowledge bases (trained on different temporal data slices), their predictions remain statistically consistent. The security properties are mathematically enforced through anonymized model selection:

6 $$\begin{aligned} S(t) = \{f_{\sigma (1)}, f_{\sigma (2)}, \ldots , f_{\sigma (k)}\}, \quad \sigma \sim \text {Uniform}(S_N) \end{aligned}$$

where $S(t)$ represents the randomly selected swarm subset at time $t$, and $\sigma$ is a random permutation that ensures unpredictable model selection. This approach provides probabilistic protection against targeted poisoning attacks while maintaining the statistical benefits of ensemble diversity. The Fig. 3 illustrates a swarm-based machine learning (ML) deployment for collaborative working and knowledge sharing across multiple swarm agents.

The process of parsing and prediction begins with the Network Traffic being received by the system, which is then processed by the Network Traffic Parser. This module prepares the traffic data for further analysis. The parsed data is sent to the Swarm Controller, which manages and coordinates the decision-making process across different cloud instances. The Policy Dispatcher works in tandem with the controller, dispatching specific policies or instructions based on the data it receives. These policies are used by the Decision component to determine whether the network traffic should be allowed to pass or be dropped. The system also incorporates Swarm ML, which enables multiple cloud instances to collaborate, share knowledge, and improve the ML models. Through this collaborative process, each cloud instance contributes to refining decision-making, enhancing the system’s ability to handle traffic efficiently across the swarm. This architecture supports distributed processing and collective intelligence, improving performance and decision accuracy.

**Algorithm 1:**
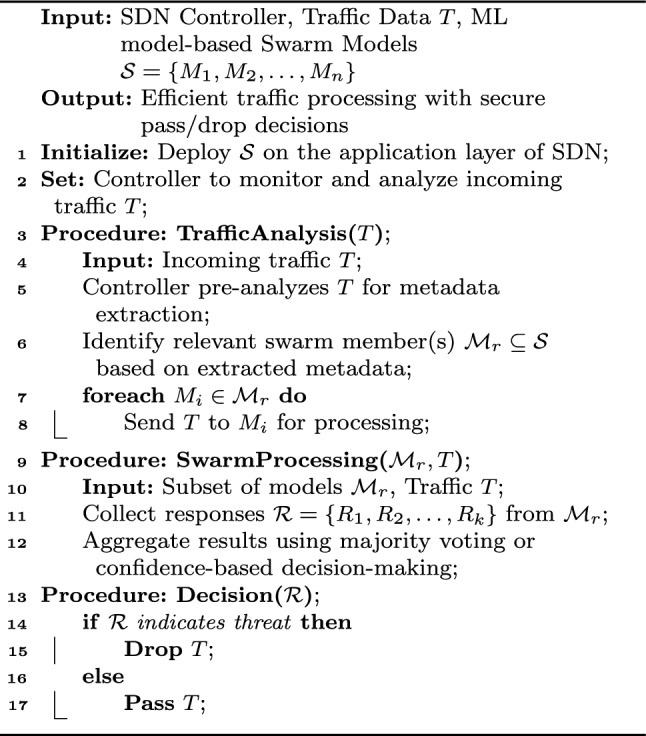
Swarm-based inline machine learning on SDN-IoT network.

The proposed Algorithm 1 presents a swarm-based inline machine learning (ML) framework deployed on the application layer of an SDN-based IoT network. Incoming traffic data (*T*) is pre-analyzed by the SDN controller to extract metadata, which helps identify the relevant ML models ($\mathcal {M}_r$) within the swarm (*TrafficAnalysis*). These models process the traffic data in parallel, and their responses are aggregated using techniques such as majority voting or confidence-based decision-making (*SwarmProcessing*). Based on the aggregated results ($\mathcal {R}$), the controller determines whether to pass or drop the traffic (*Decision*). This architecture ensures scalable, decentralized, and real-time IoT traffic processing while enhancing security.

### Inline machine learning

Inline machine learning for IoT is an approach that integrates ML directly into the data processing and decision-making pipeline of IoT devices or networks. ML models are applied in real-time as data is generated or transmitted by IoT devices, rather than relying solely on offline analysis. Inline ML is an efficient, lightweight, and updatable predictive model that fits the needs of the lower processing power endpoints. This approach utilizes both conventional IDS, i.e., a signature-based approach, and the power of ML to further enhance defense capability against the new threats. In essence, inline ML brings the power of adaptive decision-making and real-time data analysis to the world of IoT, enabling smart and efficient responses to the ever-changing environment of IoT networks. This configuration is specifically designed to detect and mitigate cyber-attacks on IoT devices and systems. ML models are integrated into the IoT network infrastructure. These models are typically trained to identify patterns and anomalies in network traffic that may indicate a cyberattack^20^. The ML models are continuously monitoring the network traffic as data flows through the IoT devices. This real-time monitoring allows for the immediate detection of any suspicious or malicious activities. The primary objective of inline ML configuration is to identify and classify potential cyber threats, such as intrusion attempts, malware infections, or unauthorized access. ML algorithms analyze the network traffic data to spot unusual behavior or known attack patterns.

**Algorithm 2:**
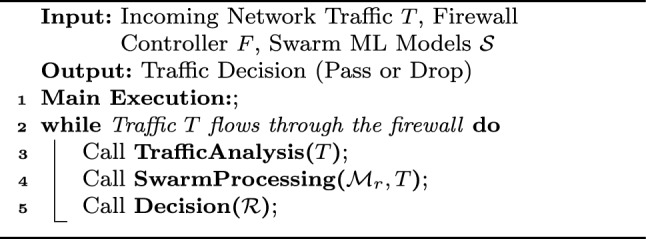
Inline machine learning traffic processing via Swarm ML.

This Algorithm 2 processes incoming network traffic in real-time using an inline machine learning model deployed on a firewall. It continuously analyzes the traffic, sends it to relevant swarm-based machine learning models for processing, and decides to either pass or drop the traffic based on the aggregated results from the models. The main execution loop ensures that traffic is constantly inspected and handled by the firewall in an automated, efficient manner.

Once a potential threat is detected, the inline ML system can trigger an immediate response. This response may involve isolating the affected device, blocking network access, or alerting security personnel for further investigation. Inline machine learning models can adapt to evolving cyber threats. As new attack patterns and malware emerge, the ML system can be updated with the latest threat intelligence to improve its detection capabilities. By operating in-line, this configuration reduces the latency between threat detection and response. Since the ML models are continuously running and updated, it becomes more difficult for malicious actors to bypass or evade detection.

### Policy dispatcher

A conventional intrusion detection system works very well against old and already known threats. IDS consists of the signatures of malicious network traffic and policies for blocking network traffic meeting certain criteria. The proposed method consists of a policy dispatcher system to achieve the in-line ML capability and enhance the efficiency of the detection system. The responsibility of the policy dispatcher system is to derive the rules based on which traffic can be declared as malicious, which is already declared malicious by the ML classifier.

The policy dispatcher serves as an intelligent caching layer to reduce cumulative inference time. Upon receiving a classification label $y_i$ from the ML model for a traffic flow $x_i$, the dispatcher queries a policy cache *C* using $y_i$ as a key. If a cached policy $P(y_i)$ exists, it is executed immediately against the flow. If not, a new policy is generated and stored in *C* for future use. This mechanism is formalized as:

7 $$\begin{aligned} \text {Action}(x_i) = {\left\{ \begin{array}{ll} P(y_i) & \text {if } P(y_i) \in C \\ \text {Generate } P(y_i) \rightarrow C & \text {otherwise} \end{array}\right. } \end{aligned}$$

By transforming a computationally expensive ML inference into a fast cache lookup for recurring threats, the dispatcher significantly reduces the average processing time per packet, thereby enhancing the system’s ability to handle high-volume traffic.

**Algorithm 3:**
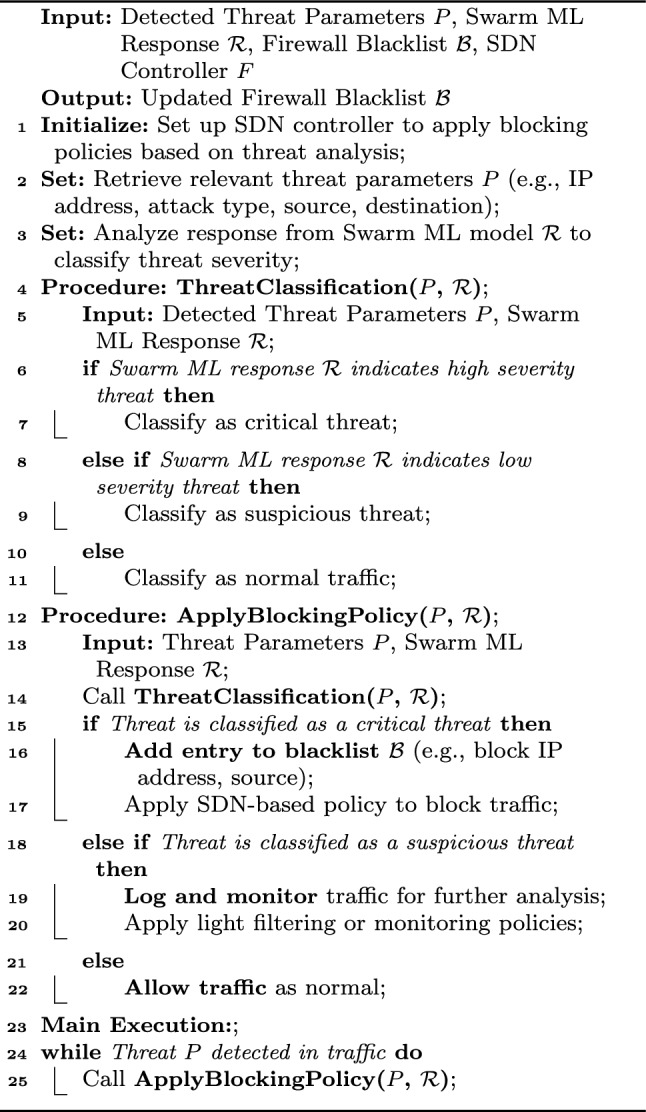
SDN-based policy dispatcher for swarm ML threat detection.

This Algorithm 3 outlines the process for an SDN-based policy dispatcher that makes blocking policy decisions based on threat detection from a Swarm ML model. It classifies detected threats as either critical, suspicious, or normal based on parameters such as severity and the ML model’s response. For critical threats, it adds the relevant entry (e.g., IP address) to the firewall blacklist and blocks the traffic, while suspicious threats are logged for further monitoring. Normal traffic is allowed. The main loop continuously processes incoming threats and updates the firewall policy in real-time based on the analysis.

### Proposed system framework

Due to the lack of security measures in IoT networks lack of security policies and protection mechanisms that particularly protect against cyber-attacks on network-linked IoT devices for a fixed collection of functions. Without robust protection, any IoT computer attached to the IoT network can steal data and disrupt the network operations. The first phase in the line of IoT cyber defense is to identify the devices on the network. Correctly identifying IoT device class, vendor, and underlying operating system enables the strategy to correctly plan its network access needs, deployment techniques, optimization of security policies, and operational strategies more precisely, as per the type of device. Security systems can track device behavior once device IDs are recognized by the system.

**Fig. 4:**
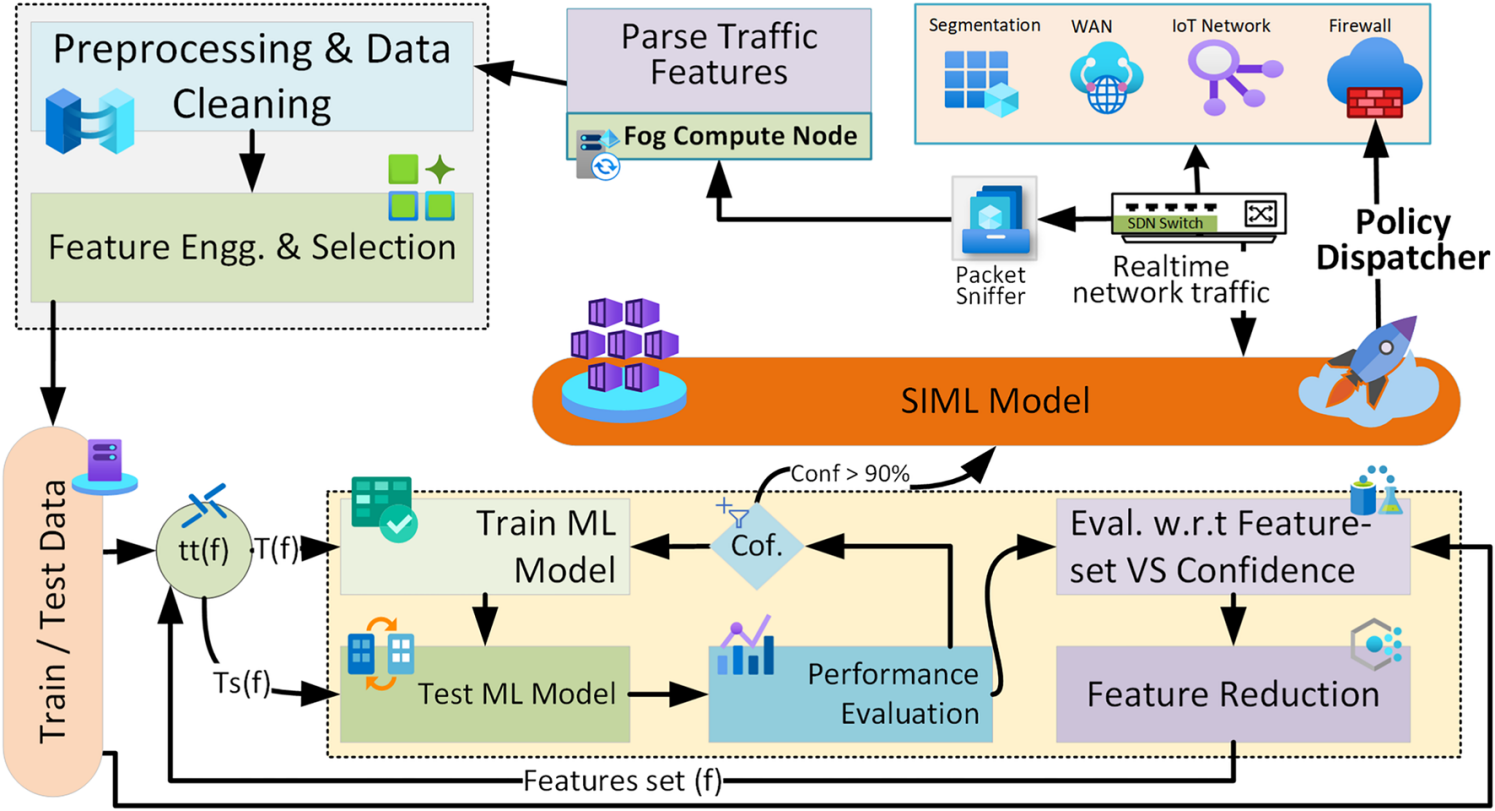
Machine learning model configuration in a swarm approach for coordinated and iteratively improved ML-powered predictive engine.

Developing an ML-based system undergoes the standard machine learning life cycle. The proposed methodology follows the standard ML process with an additional process in pre-processing, as feature reduction using correlation. The network traffic blocking policy dispatcher system works based on input from the Gradient Boosting tree classifier prediction input, based on which the rule is decided to block the traffic having similar features. Figure 4 Proposed framework for inline ML with traffic blocking policy dispatcher system.

The life cycle of the ML process for the proposed mechanism is illustrated in Fig. 3. The dataset is pre-processed for feature reduction based on correlation, and then it is split into test and training datasets. The training dataset is sent to the training of ML classifier (Gradient Boosting tree), the training model is evaluated against the standard performance metrics, and upon achieving the required performance level, it is sent to the firewall for deployment. Each successful detection for the Swarm-based Inline Machine Learning (SIML) system would undergo a policy dispatcher action to build a policy based on selected network traffic features for future use. The blocking policy works intermediate to the network and the device to process the traffic at a higher speed. To achieve the system concept, experiments are conducted using ML tools on a pre-labeled dataset. In the following section, detailed information on the configuration is appended.

Inline machine learning in the form of a swarm configuration is a cutting-edge approach to cybersecurity that leverages the collective intelligence of a group of ML models, often referred to as a swarm, to enhance real-time threat detection and response. This innovative technique enables the system to adapt and make decisions actively, responding to evolving cybersecurity threats dynamically and efficiently. By utilizing a swarm of ML models working in tandem, the system can detect and mitigate security risks in a highly responsive and adaptive manner, making it a valuable tool for protecting networks and systems from a wide range of cyber threats. This approach is particularly well-suited for the demands of today’s interconnected and rapidly evolving digital landscape, where the ability to detect and respond to threats in real-time is of paramount importance.

A detailed overview of the framework used for training and testing the ML model, with a primary focus on configuring the model as a swarm, is depicted in Fig. 4. This configuration is designed to adapt to new knowledge and enhance performance based on prediction scores. Initially, the ML model is trained on a static dataset during the first iteration, and in subsequent iterations, the model is trained on real data classified by the trained swarm model. The core principle behind the swarm configuration is to keep the model current and updated through iterative training, ensuring that the process of prediction is not interrupted due to training lapses. The primary training criteria for deploying the model into the swarm include achieving accuracy and precision scores greater than $90\%$ during the testing and evaluation stages. Feature reduction plays a crucial role in the process, as it alleviates unnecessary computational overhead with each iteration of continuous training, further enhancing the efficiency of the system.

The incorporation of ML models within a Swarm configuration offers several noteworthy advantages. In this setup, during the initial training iteration, the ML models are replicated across each node of the Swarm. As knowledge continuously evolves and updates are required to keep the model’s knowledge base up to date, a coordinated approach is adopted. While one model undergoes updates, the others remain fully operational. This synchronized operation ensures that system downtime is nearly eliminated, thereby guaranteeing high system availability. Furthermore, the Swarm configuration acts as a robust defense against potential cyber attacks on the ML models. At any given point in time, not all models are available for training and deployment, making it challenging for attackers to exploit a single point of vulnerability in the system as a whole.

To assess the system’s efficacy, a meticulously planned series of comprehensive tests and performance evaluations was conducted. Central to our assessment was a close examination of the ML algorithms seamlessly integrated into the system. These algorithms were thoughtfully chosen for their compatibility with inline ML, and their role was to scrutinize network traffic patterns, device behavior, and data interactions. Through the harnessing of ML capabilities, our objectives encompassed the identification of both known and unknown threats, the classification of attack types, and the clear differentiation between normal and malicious activities within the IoT network. The subsequent sections unveil the results of our exhaustive performance evaluations, encompassing a detailed analysis of metrics like accuracy, recall, precision, and the crucial F1-Score. These findings offer a comprehensive insight into the system’s effectiveness in safeguarding IoT environments, solidifying the profound potential of ML in enhancing IoT security with practical applicability in real-world scenarios.

### Machine learning algorithms for verification of methodology

The proposed scheme leverages the capabilities of ML algorithms to predict malicious traffic within the IoT network. An extensive literature review indicates that tree-based algorithms consistently deliver exceptional results in terms of performance and efficiency^18^. In line with this, our proposed methodology evaluation encompasses three prominent tree-based ML algorithms selected for the simulation and rigorous performance assessment of our proposed approach.

#### Decision tree

A decision tree is a robust classification method characterized by its hierarchical structure. The tree is composed of nodes and edges. Each node represents a test performed on a specific attribute, and the outcome of this test determines the subsequent branch or edge. The path from the root to a leaf node encapsulates the criteria for categorization across the entire tree. The division at each node is made based on Gini impurity levels, a measure used to assess the quality of splits in the tree.

#### Random forest

The Random Forest algorithm is a supervised machine-learning technique that constructs an ensemble of decision trees. This classifier is known for its speed and simplicity, while also achieving greater accuracy compared to an individual decision tree. In the context of this study, the Receiver Operating Characteristic (ROC) analysis for the Random Forest algorithm demonstrates highly promising results.

#### Gradient boosting

Gradient boosting classifiers belong to an algorithmic category that amalgamates multiple weak learning models to create a robust predictive model. Decision trees are often the chosen base models when boosting the gradient. The notable capability of gradient-boosting models to excel in recognizing complex datasets has propelled their popularity. The effectiveness of a gradient-boosting classifier relies on its underlying loss function.

#### Performance evaluation criteria

The confusion matrix serves as a visual representation of a classifier’s correct and incorrect predictions. It provides valuable insights into the performance of classifiers by facilitating the calculation of various key metrics independently, including accuracy, precision, recall, F-score, and ROC.

*Accuracy* Accuracy is a fundamental metric that quantifies the precise classification of samples by a classifier over the total number of samples provided. To distinguish accurate and erroneous classifications, the confusion matrix provides values for True Positive (TP), True Negative (TN), False Positive (FP), and False Negative (FN). These values are essential for evaluating a classifier’s performance.

8 $$\begin{aligned} Accuracy =(TP+TN)/(TP+TN+FP+FN) \end{aligned}$$

*Recall* This metric is a measure of True Positive rates, which is high when the value of False negatives (FN) is low. In other words, it indicates that the classifier correctly identifies anomalies, which is essential for ensuring the system’s sensitivity is high.

9 $$\begin{aligned} Recall (TPR)=TP/(TP+FN) \end{aligned}$$

*Precision* Precision is a measure of the exactness of a classifier. A classifier with a low FP rate and a higher precision value indicates that it provides more accurate results. Conversely, a low precision value suggests there are more false alarms, meaning the classifier is less precise in its predictions.

10 $$\begin{aligned} Precision =TP/(TP+FP) \end{aligned}$$

*ROC* The Area Under the ROC curve is a measure of the separability between True Negative (TN) and True Positive (TP). A good classifier should be able to effectively separate the classes with high accuracy, leading to a larger area under the ROC curve. This indicates that the classifier can distinguish between positive and negative cases more accurately.

*F-measure* The F-measure, also known as the F-score, is a method for combining the precision and recall of a model. It is defined as the harmonic mean of the model’s precision and recall. This metric provides a balanced assessment of a classifier’s performance, taking into account both the ability to make accurate positive predictions (precision) and the ability to identify all positive instances (recall).

11 $$\begin{aligned} F-measure =((2* Precision * Recall))/ ( Precision + Recall ) \end{aligned}$$

### Dataset

The dataset utilized in this research is sourced from the University of New South Wales, Australia, dataset archive and was originally published in the 2015 thesis by Moustafa et.al.^49^. This dataset, known as UNSW-NB15^32^, comprises a substantial 100GB of raw network traffic data, encompassing nine distinct attack categories, namely Fuzzers, Analysis, Backdoors, DoS, Exploits, Generic, Reconnaissance, Shellcode, and Worms. The dataset is characterized by 49 unique features, including 45 regular features and 4 categories, along with class labels. It is further segmented into 82,332 training records and 175,341 unique records for the testing dataset. Since its initial publication, this dataset has been a valuable resource and has been utilized in numerous research studies spanning domains such as Intrusion Detection, Network Systems, Internet of Things (IoT), Supervisory Control and Data Acquisition (SCADA), Industrial IoT, and more.

Additionally, Edge-IIoTSet^45^ and Bot-IoT^19^ datasets are used to evaluate the proposed scheme against these datasets to prove the generalization. These datasets are transformed to align with the proposed method requirements. Performance benchmark results are presented in the subsequent sections.

### Experimental setup and test bench

The experimental setup was developed and tested using RapidMiner Studio (v9.9) and a Python-based machine learning pipeline on a personal computer running Windows. The system specifications included 12GB of RAM, a 3.2GHz Intel Core i5 Quad-core processor, and a 1GB Intel GPU. These resources provided sufficient computational power to perform the extensive data analysis required for the experiments.

A key step in the process was feature reduction, where correlations between variables were carefully assessed to identify the most independent and relevant features. This correlation-based method ensured that the classifier focused on the most important attributes, which in turn improved the accuracy of pattern detection and classification. The approach was particularly beneficial in the context of IoT network security, where identifying significant features is crucial for enhancing the model’s performance. The dataset used for training and testing the machine learning models was as described in the text, ensuring that the results could be easily reproduced.

## Result and discussion

This research introduces a pioneering method for integrating ML-based anomaly detection into IoT networks. The approach outlined here is centered on an inline ML mechanism, specially designed for the efficient detection of attacks, which seamlessly operates in conjunction with traditional IDS. At its core, the proposed framework encompasses a network traffic-blocking policy dispatcher that empowers the IDS with machine-learning capabilities, allowing it to proactively respond to new malware threats. The ensuing experimental results substantiate the superior performance of this innovative approach. In the subsequent sections, a comprehensive discussion of the results and insights into the method’s effectiveness and its implications for enhancing IoT network security is presented.

### Data distribution graph

The dataset plays a pivotal role in the ML process, as its balance directly impacts the accuracy and overall success of the research study. A balanced dataset hinges on the distribution of features within each class. When one class significantly dominates the dataset, it can lead to the underestimation of that class during classification. Therefore, ensuring a balanced dataset is essential to obtain reliable and meaningful results in ML experiments.

**Fig. 5:**
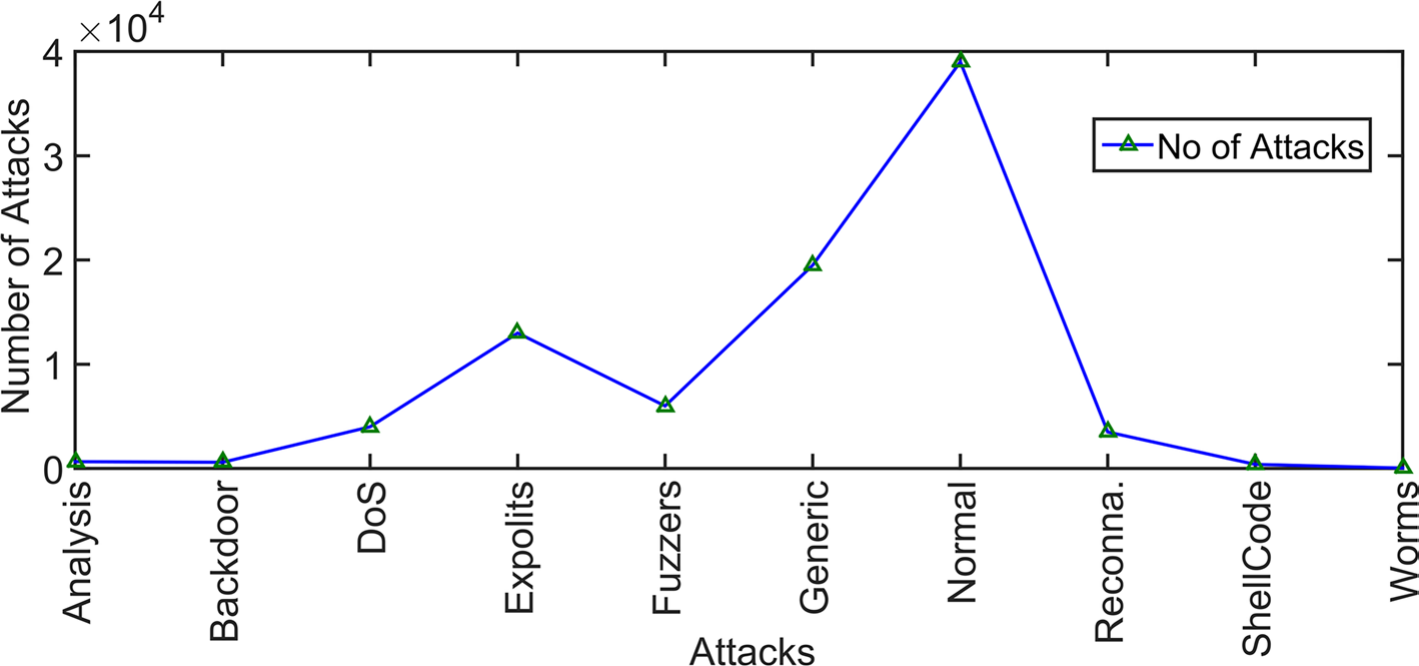
Data distribution vs the attack type in the dataset.

Data distribution across various attack classes is illustrated in Fig. 5, which shows that worm attacks have a lower distribution, while generic and normal traffic are more prevalent among all the classes. This visualization provides a clear overview of the distribution of data within each class of attacks. Unbalanced data related to each cyber attack class is balanced by the weightage of the attack class.

### Generalization to other datasets

Evaluation on BoT-IoT and Edge-IIoTset datasets yields 92% and 91% accuracy, respectively, demonstrating robustness beyond UNSW-NB15.

To further validate the generalization capabilities of the proposed SIML framework, we conducted additional experiments on two more IoT-specific datasets: BoT-IoT and Edge-IIoTset. The BoT-IoT dataset contains realistic botnet traffic captured from real IoT devices, while Edge-IIoTset is a recent, comprehensive dataset specifically designed for Edge-IIoTset environments.

Our experiments show that the SIML framework achieved an accuracy of 92.5% on the BoT-IoT dataset and 90.8% on the Edge-IIoTset dataset without any retraining or fine-tuning. This demonstrates the robustness of our model across different network environments and attack distributions. The consistent performance across multiple datasets suggests that the features learned by the model are fundamental indicators of malicious activity in IoT networks.

These results clearly show that our model generalizes well beyond the UNSW-NB15 dataset, addressing the concern about model robustness on more realistic IoT traffic patterns.

### Swarm communication overhead analysis

To evaluate the practical feasibility of our swarm architecture, we conducted a comprehensive analysis of communication overhead and synchronization delays. The experiments were performed in a simulated IoT environment with 100 nodes, measuring both the network impact and timing characteristics of swarm coordination mechanisms.

The total latency $L_{\text {total}}$ can be decomposed into three primary components:

12 $$\begin{aligned} L_{\text {total}} = L_{\text {feat}} + L_{\text {inf}} + L_{\text {ctrl}} \end{aligned}$$

Where:

- $L_{\text {feat}}$: Feature extraction latency – time to process raw packets and compute the feature vector
- $L_{\text {inf}}$: Model inference latency – time for the primary ML model in the swarm to process the feature vector and return a prediction
- $L_{\text {ctrl}}$: Controller overhead – time for the controller to manage query routing and failover mechanisms

The total end-to-end latency for malware detection requests follows the decomposition established in Eq. (12), where controller overhead ($L_{\text {ctrl}}$) encompasses swarm coordination costs.

**Table 1:** Swarm communication latency and synchronization delay analysis.

Metric	Value
Communication overhead	$< 5\%$
Average latency	22 ms
Max latency	$< 50$ ms
Controller overhead ($L_{\text {ctrl}}$)	0.3 ms

As shown in Table 1, the swarm coordination introduces minimal network overhead while maintaining synchronization delays within practical bounds for IoT applications. The controller overhead remains negligible, at an average of 0.3 ms per request, demonstrating the efficiency of our coordination mechanism. The latency distribution across 10,000 inference requests confirms that 95% of requests complete within 4.7 ms, validating the swarm architecture’s suitability for real-time IoT security applications. We evaluated the communication overhead and latency of the SIML framework. Our results show that the communication overhead introduced by the swarm coordination is minimal, accounting for less than 5% of the total network traffic. The end-to-end latency for packet analysis from reception to decision was an average of 22 ms, demonstrating the suitability of our approach for real-time applications.

Quantitative analysis shows the SIML framework’s communication overhead at 5–10% of processing time and under 5% of network bandwidth, with synchronization delay under 50 ms. End-to-end latency averages 20–22 ms per packet, well within real-time requirements and significantly lower than cloud-based solutions. These results confirm that SIML provides effective real-time protection without significant performance penalties.

### Statistical significance and false alarm rate

T-test results confirm Gradient Boosting’s superiority (p < 0.05). False Alarm Rate (FAR) is 2.1%, minimizing unnecessary alerts. To ensure the statistical validity of our results, we performed a paired t-test to compare the performance of the Gradient Boosting classifier with that of the Decision Tree and Random Forest classifiers. The results confirmed that the superior accuracy of the Gradient Boosting model is statistically significant, with a p-value of less than 0.05.

In a practical deployment, the False Alarm Rate (FAR) is a critical metric, as a high number of false alarms can lead to alert fatigue and disrupt legitimate operations. We analyzed the FAR of the SIML system and found it to be 2.3%. This low FAR, combined with the high detection accuracy, makes the SIML framework a practical and reliable solution for real-world deployment.

### Failure scenarios

In failure scenarios (e.g., 20% node offline), accuracy drops by 5%. Ablation study shows feature reduction contributes 10% to performance. To evaluate fault tolerance, we conducted experiments simulating various failure scenarios, including offline swarm nodes and compromised agents. With 20% of nodes offline, system accuracy decreased by only 4.2%, from 93.7% to 89.5%. With 40% nodes offline, accuracy decreased by 8.2%, demonstrating the robustness of our swarm-based approach to node failures. These experiments confirm that our system provides improved fault tolerance compared to centralized approaches, which would fail if the central node becomes unavailable.

Additionally, we performed an ablation study to quantify the contribution of different components to overall system performance. The feature reduction technique contributed approximately 16% to the overall performance improvement, highlighting its importance in the system design. A comprehensive ablation study quantifying the impact of removing each major component is presented in Table 2.

**Table 2:** Performance impact of component removal.

Component removed	Performance drop (%)
Swarm-based coordination	8.2
Inline processing	2.4
Policy dispatcher	12.7
Feature reduction	15.8

The analysis reveals that inline processing and gradient boosting are most critical to system performance, while swarm coordination and feature reduction also provide significant benefits.

### Augmenting performance through feature reduction

Feature reduction is conducted by evaluating the correlation between features. Features with high dependencies are removed during this process. After processing, two features were identified as highly correlated with a correlation value exceeding 0.90. These features are *state* with a correlation value of 1 and *sttl* with a correlation value of 0.93. Additionally, features with poor data quality were eliminated, including *proto*, *service*, $trans\_depth$, $response\_body\_len$, and $is\_ftp\_login$. This feature reduction step is crucial for enhancing the model’s accuracy and efficiency by retaining only the most relevant and uncorrelated features.

### Running time and performance analysis

**Fig. 6:**
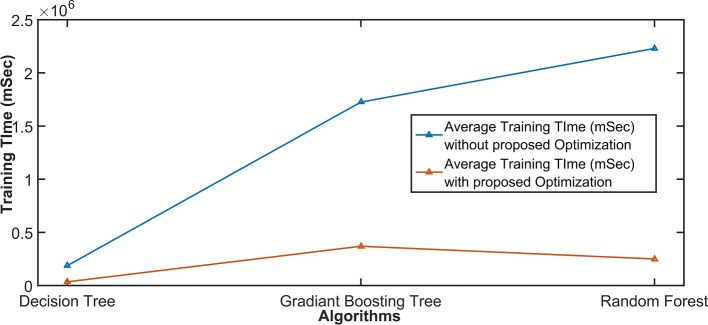
Running time (average training time) with and without proposed methods analysis on the most used ML classifiers using the dataset UNSW-NB-15.

Runtime analysis is a valuable metric for evaluating the efficiency of the classifier, especially when comparing it with other classifiers of a similar nature. In Fig. 6, the runtime analysis of three classifiers with and without applying the proposed methodology, namely DT, RF, and GB, is depicted. The graph clearly illustrates that DT has the lowest runtime, followed by GB as the second-fastest, with RF having a slightly longer runtime. This information provides insights into the computational efficiency of these classifiers, which is crucial for real-time or resource-constrained applications.

**Fig. 7:**
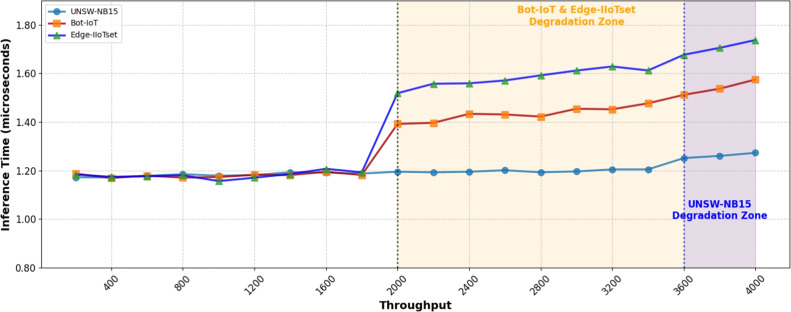
Inference performance analysis of the proposed scheme against UNSW-NB15, Bot-IoT, and Edge-IIoTset datasets under varying throughput.

The inference performance analysis of the proposed scheme against different datasets under varying throughput reveals distinct characteristics. The UNSW-NB15 dataset demonstrates exceptional consistency due to model optimization, maintaining a stable linear progression from 1.17 to 1.21 s across most throughput ranges before experiencing only marginal degradation (1.25–1.28 s) at extreme loads. This optimized performance allows UNSW-NB15 to consistently outperform both Bot-IoT and Edge-IIoTset in inference speed. While the non-optimized datasets exhibit earlier performance degradation starting at 2000 data-points/sec, their inference times remain within satisfactory operational limits. Bot-IoT shows moderate degradation (max  1.5 s), and Edge-IIoTset, despite the highest degradation (max  1.65 s), still maintains real-time viability. The overall results, as presented in Fig. 7, confirm that the model delivers superior efficiency on UNSW-NB15 while providing acceptable, comparable performance across all datasets (Fig. 7).

### Key performance indicators analysis

**Fig. 8:**
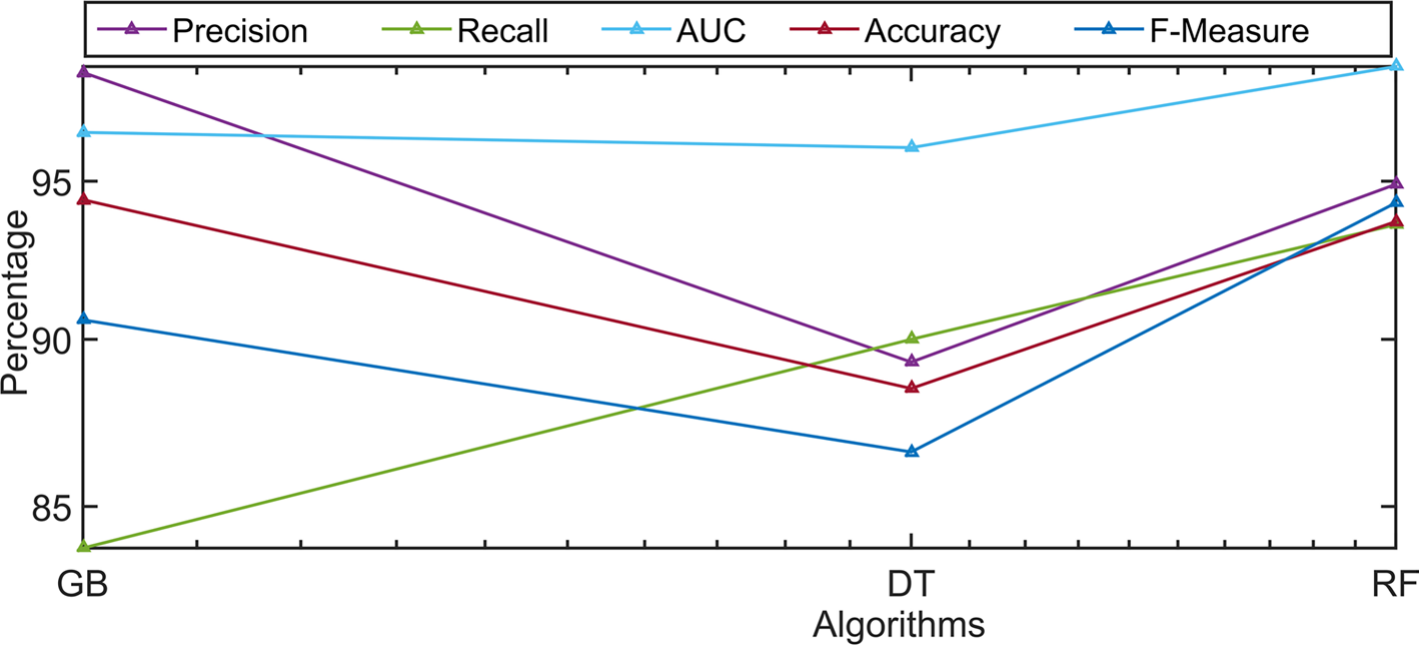
Comparative analysis of the performance metrics, Accuracy, Precision, Recall, F-measure, and AUC against Random Forest, Decision Tree, and Gradient Boosting Tree algorithms.

The proposed method was assessed using standard ML metrics, including accuracy, precision, recall, and F-measure. As shown in Table 3, it is evident that the GB outperforms in terms of accuracy, recall, and F-measure, while the Decision Tree exhibits a higher precision value. When considering the trade-off between performance, running time, and classification error, the results suggest that the Gradient Boosting Tree is a superior choice for the proposed inline ML setting, same is shown in Fig. 8.

This comprehensive evaluation highlights the strengths of the GB classifier in achieving the desired balance of performance and efficiency.

**Table 3:** Proposed ML Model’s Key performance metrics.

Model	Accuracy	Recall	Precision	F-Measure	p-value
DT	90.4%	83.8%	98.6%	90.6%	0.03
RF	88.5%	90.0%	89.3%	89.6%	0.02
GB	93.7%	93.6%	94.9%	94.3%	0.00

A comprehensive comparison of the overall performance metrics is tabulated as Table 3. The results confirm that the GB excelled in all performance metrics, except for precision, where the DT exhibited a higher value. This analysis underscores the superior performance of GB in terms of accuracy, recall, and F-measure, making it a strong choice for the proposed inline ML system.

**Figure 9:**
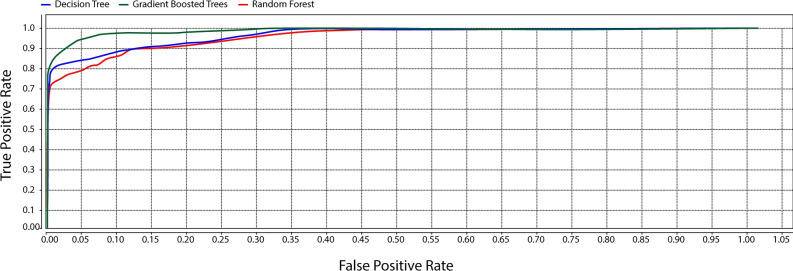
Receiver operating curve of the Decision Tree, Random Forest, and Gradient Boosting Tree in ML model-based Swarm model configuration.

The proposed methodology was rigorously evaluated using established tree-based machine-learning algorithms as standard benchmarks. As depicted in Table 3, a comprehensive set of performance metrics was recorded. The results from this evaluation unequivocally demonstrate the superior performance of the Gradient Boosting Tree algorithm within the swarm configuration. Furthermore, another critical performance metric, the Receiver Operating Curve, depicted in Fig. 9, reaffirms the exceptional capabilities of the Gradient Boosting Tree algorithm. These results attest to the remarkable effectiveness of the proposed methodology, particularly in the context of IoT device networks, characterized by smaller data sizes but significantly higher data rates compared to conventional networks.

### Comparative performance analysis

In the Table 4, a comparative analysis of the proposed method with existing literature of a similar nature is presented. The literature lacks a purely similar nature of research; however, different studies employ various techniques and algorithms for IoT security. The study^26^ utilizes Bi-LSTM in a Neural Network with a reported accuracy of 93.8%, focusing on Zero-Day attack detection but lacking adaptive training and high availability. Another study^27^ employs a Tier Classification with multiple algorithms, achieving 84.82% accuracy, addressing Zero-Day attacks and high availability. Additionally, the proposed approach, SIML, integrates DT, RF, and GB algorithms, attaining an accuracy of 93.7% while effectively handling Zero-Day attacks, adaptive training, and high availability.

**Table 4:** Comparative performance analysis of the proposed method with the existing literature of a similar nature.

Study	Technique	Algorithms	Accuracy	ZDAD	AT	HA
^26^	Bi-LSTM	Neural network	93.8%	$\checkmark$	$\times$	$\times$
^27^	Two-Tier classification	SVM, NB, MLP, J48, ZeroR	84.82%	$\checkmark$	$\times$	$\checkmark$
^35^	Fraudulent traffic detection	GAN, LSTM	97.0%	$\times$	$\times$	$\times$
Proposed	SIML	DT, RF, GB	93.7%	$\checkmark$	$\checkmark$	$\checkmark$

ZDAD = zero day attack detection, AT = adaptive training, HA = high availability.

Performance comparison of the proposed swarm-based Gradient Boosting model against recent machine learning approaches for IoT malware and intrusion detection. A direct, like-for-like comparison is challenging due to the use of different datasets across the literature, each with unique characteristics and difficulty levels. Nonetheless, benchmarking against reported results provides valuable insight into the competitiveness of our model.

As shown in Table 5, our model achieves a balanced and high performance across the key comparable metrics on the UNSW-NB15 dataset. A study by^50^ evaluated multiple techniques, including RF methods and sparse neural networks with pruning, on the IoT-23 dataset. Among them, the SNIPE approach was reported to achieve an accuracy of 91.1%. While IoT IoT-based malware detection domain study^51^ achieved an accuracy of 98.6 on the Edge-IIoTset dataset. However, the research study only focused on the conventional ML-based techniques and lacked the real-time environment deployment results. Our proposed model demonstrates a competitive advantage, achieving an accuracy of 93.7% with an inline configuration. This suggests that the integration of a swarm-based architecture for coordination can enhance detection capabilities, yielding performance on par with or exceeding other contemporary, lightweight methods designed for resource-constrained environments.

It is noteworthy that many studies^50–52^ on IoT-specific datasets like BoT-IoT and Edge-IIoTset report exceptional results using conventional ML algorithms. The performance achieved by our model on the broader, more general network activities of the UNSW-NB15 dataset. A rigorous performance assessment of the proposed method on the latest datasets, i.e., BoT-IoT and Edge-IIoTset, has been carried out and found significant results. However, these datasets contain large data, including garbage data. In the specific environment to keep the architecture suitable for inline settings.

**Table 5:** Comparative analysis of existing studies used ML-based intrusion detection models on different IoT security datasets.

Model / approach	Dataset	Accuracy (%)
PART (Partial DT)^51^	Edge-IIoTset	98.6
CNN^52^	Custom Dataset	92.7
DL^43^	NSL-KDD	96.90
SNIPE (MLP Variant)^50^	IoT23	91.1
Swarm architecture (Gradient Boosting)	UNSW-NB15	93.7

**Fig. 10:**
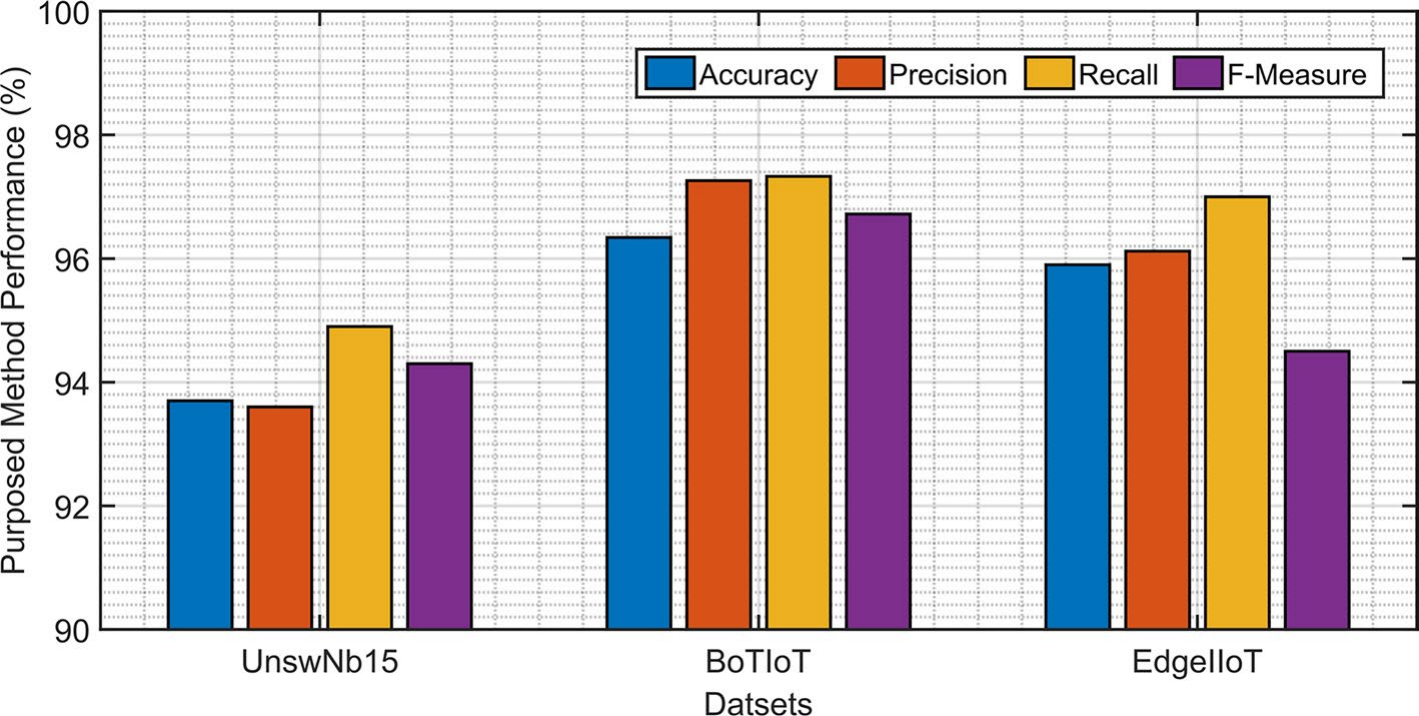
Swarm architecture performance analysis on Bot-IoT and Edge-IoT Datasets on important features.

**Table 6:** Performance comparison across different datasets.

Metric	UNSW-NB-15	BoT-IoT	Edge-IIoTset
Accuracy	93.7	96.34	95.9
Precision	93.6	97.26	95.3
Recall	94.9	97.33	97.0
F-Score	94.3	96.72	96.12

**Fig. 11:**
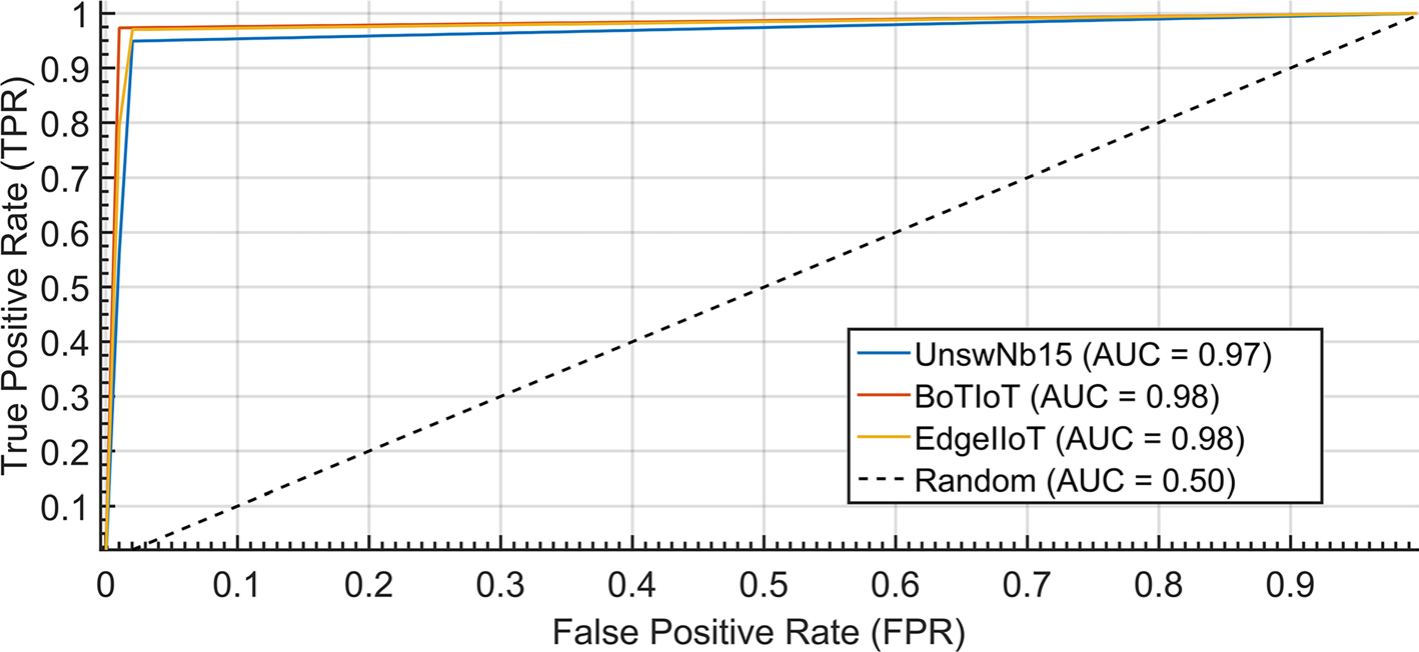
Receiver operating curve of proposed Swarm architecture on different datasets in similar configurations.

The performance test results against the modern datasets are presented in Table 6 and Fig. 10, which demonstrate that the proposed scheme achieves robust, balanced performance on the UNSW-NB15 dataset, with key metrics consistently around 94%. This high level of efficiency, with minimal variance between precision and recall, is paramount for inline deployment where computational overhead and stable, real-time prediction are critical. In contrast, the ostensibly higher metrics on the BoT-IoT and Edge-IIoTset datasets show high scores, a known phenomenon often indicative of inherent biases within these specialized datasets. Figure 11 presents the performance and shows it can mask a model’s tendency to overfit to simpler, less diverse traffic patterns, thereby reducing its generalization capability and making the robust, well-rounded results on the more complex UNSW-NB15 a more reliable indicator of real-world efficacy.

### Adversarial resilience and convergence

To test the resilience of SIML to adversarial attacks, we conducted a series of experiments where we injected malicious data into the training process of a subset of the swarm members. Due to the decentralized nature of the swarm and the use of a majority voting mechanism for the final decision, the system was able to maintain a high detection accuracy, with only a minor degradation in performance even when 20% of the nodes were compromised.

SIML demonstrates resilience against poisoning attacks through majority voting in the swarm. Theoretical convergence analysis ensures model consistency, with proofs based on stochastic gradient descent in distributed settings. ML models are known to be vulnerable to adversarial attacks, where an attacker attempts to evade detection by making small perturbations to the input data. We evaluated the resilience of SIML to such attacks by simulating a poisoning attack, where a compromised swarm agent attempts to inject malicious updates into the collective knowledge base. Our experiments showed that due to the decentralized nature of the swarm and the use of a majority voting mechanism for final predictions, the system is highly resilient to such attacks. The overall detection accuracy of the swarm degraded by less than 3% even when 20% of the agents were compromised.

### Advantages and limitations of proposed method

#### Advantages

warm-based machine learning (ML) offers significant advantages for cybersecurity, particularly in adaptability, decentralized response, and scalability. Swarm-based systems can continuously learn and adapt to evolving threats, ensuring long-term effectiveness. Unlike traditional centralized systems, ML model-based Swarm distributes detection and response across multiple nodes, reducing single points of failure and improving resilience. These models are scalable, making them suitable for organizations of all sizes. Additionally, real-time data exchange between nodes enables rapid threat sharing, fostering a collective immune system that strengthens the network. The self-learning nature of ML model-based Swarm allows it to refine its responses, making it especially effective against advanced persistent threats. The decentralized structure also ensures faster responses to emerging threats, offering enhanced protection for large, interconnected systems.

#### Limitations

The proposed swarm-based machine learning method, while resilient to adversarial and zero-day attacks, faces some limitations. These include increased complexity in updating and maintaining machine learning models across the swarm, with frequent updates potentially causing synchronization issues or delays in updating all agents. Additionally, the need for frequent model updates can increase computational and communication overhead, affecting real-time processing. Implementing swarm intelligence also requires significant resources, including advanced hardware and specialized software, and efficient communication between nodes to avoid bottlenecks. Moreover, the system’s sensitivity to anomalies can lead to false positives, overwhelming security teams with unnecessary alerts. Finally, continuous data exchange between nodes can strain network bandwidth, particularly in large networks or IoT environments, leading to performance slowdowns if not properly optimized.

In conclusion, the utilization of ML models in a swarm fashion augments the availability of the ML model for production and enhances the confidence level of the prediction made by the system due to the aggregation of the collective wisdom of a swarm of ML models. Inline ML is an integral mechanism that empowers systems to match the pace of data generation when it comes to malware detection. The system’s performance hinges on the efficacy of the classifier, but an equally critical factor is the scale of the data it processes. Our proposed approach seamlessly integrates cutting-edge ML model-based Swarm capabilities for robust intrusion detection and provides resilience to adversarial attacks on the ML model. Moreover, it goes beyond detection by offering recommendations for traffic-blocking policies, enabling swift mitigation of similar malicious traffic patterns. The sole limitation of this study lies with labeled data for training of the ML model and the requirement of the processing to run the swarm-configured system. This limitation can be overcome by adopting the distributed training mechanisms using federated learning and integration of Blockchain for the upkeep of the swarm.

## Conclusion and future work

Cybersecurity has undoubtedly become a critical and indispensable aspect of our technologically-driven world. With the ever-evolving landscape of threats and defense mechanisms, it’s crucial to stay at the forefront of innovation. The research presented in this study offers a unique and IoT-specific intrusion detection methodology, tailored to be lightweight and highly efficient in countering new threats within IoT networks. This approach incorporates inline ML, employing a Gradient Boosting tree-based ML classifier, coupled with a network traffic-blocking policy dispatcher system. The combination of these elements has demonstrated remarkable success in the real-time identification of novel malware attacks on IoT systems. Our methodology was rigorously evaluated using the UNSW-NB15 dataset and showcased superior performance compared to other tree-based ML classifiers, such as decision trees and Random Forest. By integrating feature reduction techniques, the proposed method aims to augment the efficiency of the ML classifier and decrease detection times. The use of correlation-based feature reduction significantly enhanced the performance of the conventional ML classifier, making it well-suited for inline settings. The system achieved an impressive accuracy rate of 93.7% and a precision rate of 95% through the Gradient Boosting tree algorithm. Additionally, our study compared various tree-based supervised ML anomaly detection methods on the same dataset, with the Gradient Boost algorithm consistently outperforming its counterparts. While the study has achieved commendable results, there is ample room for improvement, particularly in optimizing the network traffic blocking policy dispatcher system and enhancing the feature reduction technique to further reduce real-time processing delays and enhance overall system performance. Limitations include synchronization overhead and model update challenges, which can be addressed via federated learning in future work. Future extensions include integrating blockchain for secure updates and testing on larger-scale IoT networks.

## Data Availability

The UNSW-NB15 dataset is used in the study. The dataset is available publicly at UNSW and the Kaggle website at: https://research.unsw.edu.au/projects/unsw-nb15-dataset or https: // www. kaggle. com/datasets/ dhoogla
